# The Curability of Mammary Carcinoma

**Published:** 1898-12

**Authors:** 


### THE CURABILITY OF MAMMARY CARCINOMA.

The time was, not so very long ago, when the diagnosis of of cancer of the breast was equivalent to passing a sentence of death, and operation was performed rather with a view of adding a few months to life or of promoting euthanasia than with any expectation of permanent good results. That improvement in results should come about through improvement in the operative technique seemed little likely, for the operation is one that has belonged to coarse surgery, as its common name "amputation of the breast" indicates; and in the performance of this class of operations there have been in the past surgeons whose skill and dexterity would equal if not excel that of the best operators of to-day.

None the less it has actually happened that the operation of removing the breast has been so greatly improved of late years that the outlook for the unfortunate subjects of mammary cancer is wonderfully improved. Their condition is so far from hopeless that they have a fair prospect of complete cure, and, what is better, the percentage of success is constantly increasing as one improvement after another is added to the operation. To remove a breast properly can no longer be spoken of as a piece of coarse surgery. The amputation part of the operation is but a small piece of the work, which as developed chiefly by Halstead includes the removal not only of a liberal margin of sound skin and subcutaneous fat, but also the complete removal of the pectoral muscles with their fascia, a minute and careful dissection around the sheath of the axillary vessels for the purpose of removing all the axillary glands, and sometimes the removal of the clavicular glands as well.

Warren, of Boston, who has taken a hand in the development of the successful operation for cancer of the breast, made a communication upon the subject to the Massachusetts Medical Society at its last meeting, in which he gave the statistics of seventy-two cases that had come into his hands during the last fifteen years. Reports were obtained upon sixty-four of these cases, showing that twen y-six were alive and thirty-eight dead, two of the latter, however, having died of other disease than cancer and long after the danger limit had passed, so that in a general way it may be said that nearly half of the patients operated upon were alive when the report was made.

Of forty-one cases where the recurrences could be studied, thirty-seven showed recurrence during the first three years after operation, and making allowances for the number of cases where the return of the disease was for a time overlooked, it may be safely claimed that when at the end of three years after an operation a competent surgeon fails to find evidence of recurrence the cure is complete and permanent. Dr. Warren says that this will be found to be true ninety-nine times out of a hundred. Applying this test to his report, there are seventeen such cases, one of which died ten years after the operation of apoplexy and one six years after of cholera. The percentage of cures according to this standpoint is thirty and nine-tenths. This is for operations covering fifteen years, but if only those cases be considered which have been operated upon under the improved technique, there are twenty-two cases with eight cures, a percentage of thirty-six and three-tenths. Dowd has recently given the statistics of one hundred and ninety-nine cases with seventy-one cures, a percentage of thirty-nine and six-tenths, but there is reason to think that some selection was used in taking the cases for report.

The kind of cancer in any given case must greatly influence the prognosis. Out of the seventeen cures reported above but one was a medullar cancer. In this case there was recurrence in the pectoral region two years later; this was successfully removed three years ago and there has been no further return. The prognosis of a case must therefore depend somewhat upon the variety of carcinoma that presents itself, the scirrhus and the intermediary form between it and medullary giving the best hope of successful treatment.

In eight out of the seventy-two cases there was a history of trauma and in four a history of abscess. Retraction of the nipple was noted in fifteen instances. In practically every case the axillary glands were found to be involved, and in five affection of the supraclaviulcar glands was noted also. After one of the early operations the disease returned in a pira mammillary gland, justifying the more recent practice of making so clean a sweep as to include all glandular tissue.

After all that has been said about the importance of early operation, in sixty-eight of the cases the disease had been known to exist for an average period of more than ten months. That earlier operations will give far better percentages of success there can be no donbt, but as Dr. Warren says, there is a curious propensity on the part of the cancerous to conceal their malady, and this must ever stand in the way of the best results from operative treatment.—*Northwestern Lancet.*

